# Prevalence and epidemiological characteristics of congenital cataract: a systematic review and meta-analysis

**DOI:** 10.1038/srep28564

**Published:** 2016-06-23

**Authors:** Xiaohang Wu, Erping Long, Haotian Lin, Yizhi Liu

**Affiliations:** 1 State Key Laboratory of Ophthalmology, Zhongshan Ophthalmic Center, Sun Yat-sen University, Guangzhou, Guangdong, 510060, People’s Republic of China

## Abstract

Congenital cataract (CC) is the primary cause of treatable childhood blindness worldwide. The establishment of reliable, epidemiological estimates is an essential first step towards management strategies. We undertook an initial systematic review and meta-analysis to estimate the prevalence and other epidemiological characteristics of CC. PubMed, Medline, Web of Science, Embase, and Cochrane Library were searched before January 2015. A meta-analysis with random-effects model based on a proportions approach was performed to determine the population-based prevalence of CC and to describe the data regarding the laterality, morphology, associated comorbidities and etiology. Heterogeneity was analyzed using the meta-regression method, and subgroup analyses were performed. 27 studies were selected from 2,610 references. The pooled prevalence estimate was 4.24 per 10,000 people, making it a rare disease based on WHO standards. Subgroup analyses revealed the highest CC prevalence in Asia, and an increasing prevalence trend through 2000. Other epidemiological characteristics showed CC tended to be bilateral, isolated, hereditary and in total/nuclear morphology. Huge heterogeneity was identified across most estimates (I^2^ > 75%). Most of the variations could be explained by sample size, research period and age at diagnosis. The findings provide suggestions for etiology of CC, improvements in screening techniques and development of public health strategies.

Congenital cataract (CC), which refers to opacity of the lens detected at birth or at an early stage of childhood^1^, is the primary cause of treatable childhood blindness worldwide^2^. An estimated 200,000 children are bilaterally blind from cataracts, and many more suffer from partial cataracts that progress and cause increasing visual difficulty as the child ages^3^. Although relatively rare compared with age-related cataracts, CC tends to alter the quality of sensory information available to the child during sensitive periods of visual system development and causes irreversible visual defects^4^. Despite the great efforts made to improve the management of CC and a giant leap in surgical techniques, CC treatment is among the most difficult and cost-intensive interventions in ophthalmology, and the etiology of this condition remains largely unknown^5^. Considering its huge burden on society, especially when expressed in blindness-years, the control of CC and blindness in children is one of the main priorities of Vision 2020: The Right to Sight, the global initiative to reduce the world’s burden of avoidable blindness^6^.

Reliable estimates of the prevalence and epidemiological characteristics of CC are essential for providing clues about the mechanisms of cataractogenesis, developing effective prevention strategies and implementing public health programs. Unfortunately, because of limited public awareness and health systems for rare disease, the few large-scale epidemiological studies involve specific regions, limited populations and partial epidemiological variables. Moreover, these descriptive studies were not based on a synthesis of the evidence and results. Thus, we conducted the first systematic review of worldwide epidemiological studies on CC and estimated the population-based prevalence of CC and its main epidemiological traits, including laterality, morphology, associated comorbidities and etiology. The purpose of this study was to provide a methodologically reliable, global and current pooled prevalence of CC and to collect other major epidemiological data to shed light on the etiology of this condition and to promote the development of screening and public health management strategies related to CC.

## Results

### Summary of Included Studies

**Figure 1:**
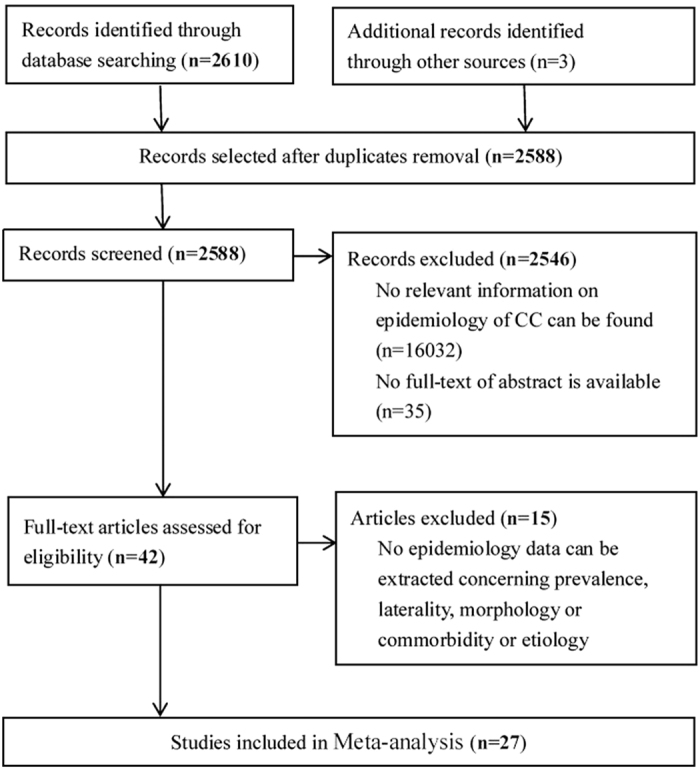
Flowchart of study selection process.

A total of 2,613 articles were initially identified. After duplicates and nonrelevant studies were removed, the abstracts of the remaining studies were reviewed, and 42 articles with potentially relevant studies were further identified in full text. Finally, 27 published studies were determined to be eligible and were included in this meta-analysis. For details, please refer to Fig. 1.

Among the 27 eligible studies published from 1983 to 2014, 17 included data on the population-based prevalence of CC (including 4 national prospective birth cohorts, 3 national surveillance/screening/case reviews, 2 regional prospective cohorts and 8 regional surveillance/screening/case reviews). Additionally, 2 hospital-based studies and 8 CC-based case reviews were included to determine estimates of the other major epidemiological characteristics. Eleven studies were from Europe, 10 were from Asia, 4 were from the USA, 1 was from Africa, and 1 was from Australia. The sample sizes of the included studies ranged from 76 to 2,616,439 children, with a combined total of 8,302,708 children in the estimate of global CC prevalence. The extracted age at diagnosis ranged from birth to 18 years of age. For more details, refer to Table 1.

### Quality Assessment

Quality assessment scores of the included observational studies are listed in Table 1, and the details for the assessment items in domains for each article can be found in Table 2. No included studies received stars from either of the two variables of NOS, namely selection of the non-exposed cohort and comparability of cohorts since comparison studies were not represented in the included articles. Out of the remaining 6 possible points, 3 studies received 6 points, 13 studies received 5 points, 8 studies received 4 points, 2 studies received 3 points and 1 study received 2 points.

### Global Prevalence Estimate of CC

**Figure 2:**
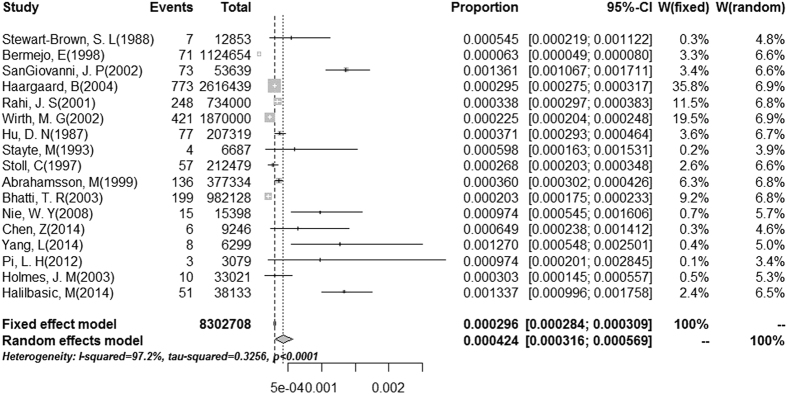
Forest plot for the prevalence of CC in population-based studies.

**Figure 3:**
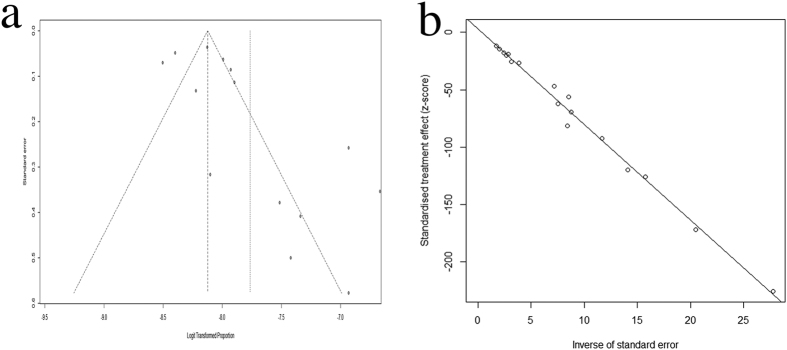
Publication bias testing for population-based CC prevalence studies. (**a**) Funnel plots. Each point represents a separate study on the indicated association. The vertical line represents the mean effect size. The points are distributed asymmetrically, indicating the existence of publication bias. (**b**) Linear regression test of funnel plot asymmetry (Egger test). The intercept indicating bias is 3.07. P-value = 0.21, indicating insignificant publication bias.

Pooled estimates of CC prevalence were calculated for 17 population-based epidemiological studies that included 8,302,708 children. Because the prevalence extracted from each study ranged from 2.2/10,000 to 13.6/10,000, logit transformation was performed on the raw prevalence data in advance. The result of the Shapiro-Wilk normality test (W = 0.93629, P-value = 0.2768) confirmed the normal distribution of the transformed sample data. The overall pooled prevalence was 4.24/10,000 (95% CI 3.16–5.69/10,000) using a random-effects model. The I^2^ statistic (97.2, P < 0.01) indicated substantial heterogeneity (Fig. 2). Publication bias was assessed by constructing a funnel plot (Fig. 3a) followed by the Egger test (P = 0.1869), and the results indicated an insignificant level of publication bias (Fig. 3b). Considering the effect of diagnosed age and included study quality on the pooled prevalence, a series of combination of studies were analyzed and the pooled prevalence ranged from 1.91/10,000 to 4.24/10,000 (Table 3).

### Source of Heterogeneity Analysis for the CC Prevalence Estimate: Meta-Regression

According to a visual inspection of the forest plot and a general analysis of the included studies’ baseline, five categorical covariates were examined as sources of potential heterogeneity. In the univariate meta-regression analyses, world region (China [developing countries] or the rest of the world [developed countries]) and study type (birth cohort or other) were not significantly associated with the CC prevalence (P = 0.085; 0.423). Significant estimates were found for the covariates of sample size (less or more than 100,000), diagnosed age (birth to 1 year old or above 1 year) and research period (before or after year 2000). The R^2^ (amount of heterogeneity accounted for) and P values for each covariate estimate were R^2^_size_ = 58.56%, P_size_ < 0.001; R^2^_age_ = 17.55%, P_age_ = 0.038; and R^2^_year_ = 37.29%, P_year_ = 0.002, respectively. A subsequent multivariate mixed-effects meta-regression model was developed based on sample size, age at diagnosis and research period, with each of these variables showing significant associations with the pooled prevalence heterogeneity. These three covariates significantly accounted for 65.41% of the heterogeneity in the CC prevalence estimate (R^2^_size+age+year_ = 65.41%, P_size+age+year_ < 0.001).

### Variations in CC Prevalence: Subgroup Analysis

**Figure 4:**
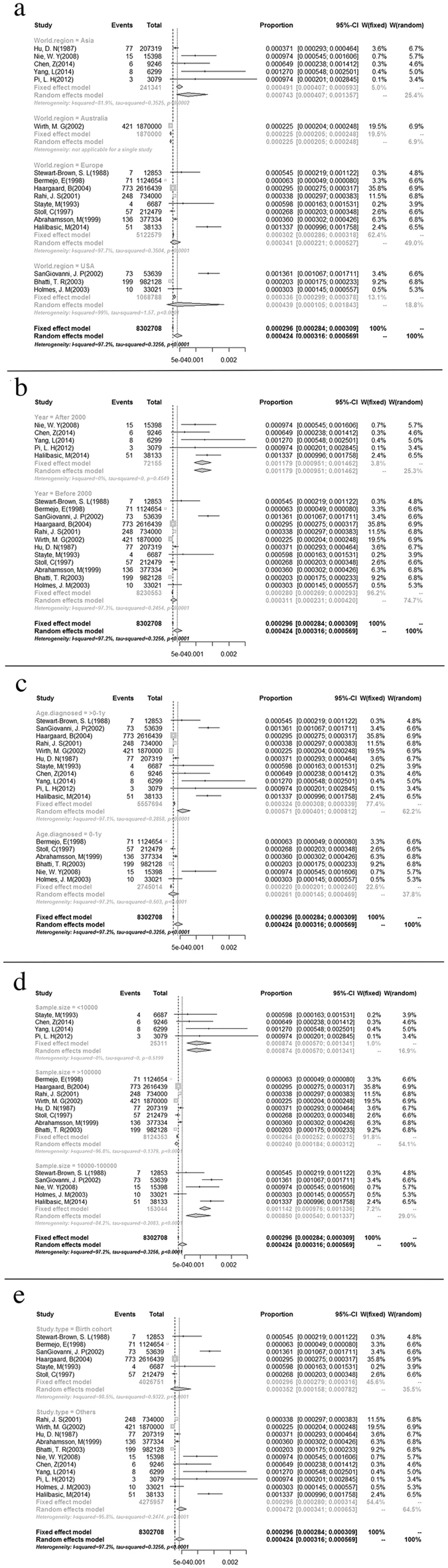
Forest plots for the subgroup analysis of population-based CC prevalence. (**a**) Forest plot of the subgroup analysis by world region. (**b**) Forest plot of the subgroup analysis by research period. (**c**) Forest plot of the subgroup analysis by age at diagnosis. (**d**) Forest plot of the subgroup analysis by sample size. (**e**) Forest plot of the subgroup analysis by study design.

The CC prevalence was further analyzed by subgroup according to world region, research period and age at diagnosis. Regarding the potential variations among world regions, the highest CC prevalence was estimated in Asia (7.43/10,000, I^2^ = 81.9%), followed by the USA (4.39/10,000, I^2^ = 99.0%), Europe (3.41/10,000, I^2^ = 97.7%) and Australia (2.25/10,000, based on a single study; Fig. 4a). Subgroup analysis by research period revealed an apparent increase in prevalence from the “before 2000” group (3.11/10,000, I^2^ = 97.3%) to the “after 2000” group (11.79/10,000, I^2^ = 0; Fig. 4b). When a subgroup analysis was performed according to age at diagnosis, the CC prevalence was higher in the “>1 year old” group (5.71/10,000, I^2^ = 97.1%) than in the “birth to 1 year old” group (2.61/10,000, I^2^ = 97.2%). In the sample size subgroups, the CC prevalence decreased (8.74/10,000, 8.50/10,000, 2.40/10,000) as the sample size increased (<10,000, 10,000–100,000, >100,000). Regarding study type, birth cohort studies (3.62/10,000) showed lower prevalence than other study designs did (4.72/10,000; Fig. 4c–e).

### Pooled Estimates of Other Major Epidemiological Characteristics

The pooled prevalence of subsets according to major epidemiological characteristics, including laterality, morphology, comorbidity and etiology, was also explored (Table 3). Bilateral cataracts accounted for 54.1% of the laterality. Regarding morphology, the three most common types of CC were total (31.2%), nuclear (27.2%), and posterior subcapsular (26.8%). According to the comorbidity reported, isolated CC, CC with ocular disorders, and CC with systemic disorders accounted for 62.3%, 22.7%, and 17.3% of cases, respectively. Regarding etiology, the pooled proportions of hereditary, nonhereditary and idiopathic CC were 22.3%, 11.5%, and 62.2%, respectively. A relatively large degree of heterogeneity was also identified across most estimates (I^2^ > 60%). Details can be seen in Table 4.

## Discussion

Our analysis provides comprehensive, current estimates of the worldwide prevalence of CC and its major epidemiological characteristics. We estimated the global CC prevalence to be 4.24/10,000, with the highest prevalence observed in Asia and an increasing trend reported through the year 2000.

### Research Contributing to Efficient Data Utilization and Comprehensive Information Integration for a Rare Disease

The prevalence of CC was estimated to be 4 to 5 patients per 10,000 children worldwide, which makes it a rare disease based on WHO (<6.5/10,000)^7^ and European (<5/10,000)^8^ standards. In contrast with common disorders, rare diseases have a much lower population prevalence, resulting in greater demands for documenting disease data and a greater reliance on accurate and comprehensive epidemiological information^9^. Unfortunately, as a result of limited public awareness and few related health systems, the few large-scale epidemiological studies have focused on specific regions, limited populations and partial epidemiological variables, with limited synthesis of the evidence and results.

Systematic review and meta-analysis provides a scientifically logical way to synthesize epidemiological data. However, the results of such methods are rarely available for rare disease with prevalence lower than 10/10,000. Possible explanations include for this lack are that extremely low prevalence variables must undergo relatively complicated transformations before they fit a normal distribution and that the high heterogeneity of the observational epidemiological studies may discount their reliability if left untreated without intensive analysis. Despite these obstacles, we provided the first ever comprehensive, worldwide estimate of the population-based prevalence of CC and its major epidemiological characteristics with the hope of providing a valuable reference for future studies on rare diseases.

### Estimates of the Global Population-Based CC Prevalence

The prevalence of CC ranged from 2.2 to 13.6 per 10,000 children in the included studies performed worldwide. The discrepancies among the studies that estimated the CC prevalence affects their reliability and the ability to compare results, and such discrepancies should be cited with regard to the following four aspects: **First,** the inclusion of CC patients was based on diverse definitions and diagnostic methods. The age at diagnosis of the patients in these studies ranged from birth to 18 years old. Holmes’ study^10^ even incorporated visually significant CC into their estimated prevalence. Despite the worldwide agreement that CC is present at birth or detected within the first year of life, the delay in detection makes it difficult to establish a diagnostic age range and is the main cause for the overestimation or underestimation of the reported prevalence. **Second,** the designs of the included studies varied from individual hospital-based cross-sectional studies to large-scale national prospective cohort studies. This difference can be partially explained by the substantial gap in medical care and medical recording systems. Most developed countries are equipped with complete referral-based structures and medical recording documentation systems that provide opportunities for high-quality, population-based prevalence studies^11^,^12^. In contrast, some developing countries, including China^13^ and India^14^, can only afford regional or hospital-based studies. **Third,** the variation in CC prevalence over time remains controversial, as these results are based on quite limited research data. Bhatti^15^, examining the CC data in Metropolitan Atlanta from 1968 to 1998, showed apparent high prevalence peaks in 1977 and 1979; yet, in Abrahamsson’s study^16^ of the population of western Sweden from 1980–1996, there was no evidence of an increase or decrease in the incidence of cataracts in the population during the study period. This inconsistency can be understood by assuming that the changing trend in CC prevalence is the result of both the increasing detection rate and the decreasing occurrence of this birth defect as health care systems develop around the world. Besides, a series of complicated biological, environmental and socioeconomic factors would be taken into consideration (including the burden of environmental toxins, infectious diseases, climate change, etc). More observations across wide periods based on different populations are needed to confirm this trend. **Fourth,** the range of prevalences among studies can be explained partially by true differences among populations from relatively isolated continents. In our study, both visual inspection and subgroup analysis by world region indicated a higher CC prevalence in Asia (or developed countries). While the underlying causes of this difference remain unknown, possible explanations include different hereditary or environmental risk factors.

### Clues from Major Epidemiological Characteristics Data

The common understanding for many years has been that roughly one-third of CC cases are inherited, one-third are associated with environmental risk factors and the remaining one-third are idiopathic^17^. However, according to the pooled proportion estimate in this study and previously reported data^18^, idiopathic CC accounts for as much as two-thirds of all CC cases. Observation and analysis of the idiopathic aspects of this condition leave room for progress in this area of research in terms of both cataractogenesis and clinical considerations.

Although bilateral CC constituted only a slightly higher pooled proportion than unilateral CC in our study, the proportions were quite different in subsets formed according to hereditary factors. As Rahi^18^ reported in a UK CC case review study, unilateral CC constitutes 56% of idiopathic CC but just 6% of hereditary CC. Similar results were seen in an Australian study by Wirth^19^. The higher proportion of unilateral CC cases with idiopathic CC indicates that this association may serve as a potential breakthrough point for exploring idiopathic CC.

The morphology subset analysis in our study indicated that total and nuclear cataracts were the two most common types of CC, which is concordant with most previously reported data^20^,^21^. In the USA, only 4 of 199 children have been classified as total cataract cases^22^. However, in the developing world, total cataracts are commonly observed in children^23^. This difference likely relates to the timing of cataract detection in the developing world. Many cataract types, if left untreated, will slowly become diffuse, total cataracts. Nuclear cataracts both the most common CC type and the type that results in the most severe visual impairment based on subjective scales^24^. Thus, clinical ophthalmologists and pediatricians should pay greater attention to the detection and treatment of this type of CC.

### Study Strengths and Limitations

The current study should be interpreted within the context of its strengths and limitations. The primary strengths of this meta-analysis are the following two aspects: **First,** we strictly followed the MOOSE guidelines for reporting systematic reviews and meta-analyses, using a critical appraisal of study quality, strict application of inclusion and exclusion criteria and up-to-date estimates using a random-effects model with logit transformed values. Heterogeneity was detected and analyzed attentively using a meta-regression model and subgroup analyses. As a result, our study can be referenced by research peers conducting meta-analyses on low-prevalence diseases. **Second,** our study was the first to include CC epidemiological information from a variety of world regions (Asia, Europe, Australia, Africa, and the USA) over a wide research period (1959–2010).

However, this review has a few limitations. **First,** in large continental regions such as Asia, there were insufficient studies to provide representative estimates for the region. For instance, in Asia, the epidemiological data were derived exclusively from China and India. **Second,** given the limitations of the collected data, we could only provide pooled proportions for each separate epidemiological characteristic. Thus, more studies are needed to focus on the interrelationships between these factors. **Third,** the heterogeneity of the included studies in terms of study design, world regions, diagnosed age and other unknown factors made it difficult to achieve valid and stable meta-analysis results despite the use of a standardized analysis process.

In conclusion, our study provides estimates that reflect the present global burden and epidemiological traits of CC. The findings of this study provide directions for further studies in this area and will be useful for the design of CC screening, treatment, and related public health strategies.

## Methods

### Search methods for identifying studies

This review followed the Meta-Analysis of Observational Studies in Epidemiology (MOOSE) guidelines for reporting systematic reviews and meta-analyses^25^. We performed a literature search of the electronic databases PubMed, Medline, Web of Science, Embase, and the Cochrane Library up to January 2015. We also manually checked the reference lists of all retrieved studies, review articles, and conference abstracts using electronic searches. In our literature search, we included a combination of keywords, such as congenital cataract, prevalence, epidemiology, population, and survey, in the form of title words or medical subject headings. For details, please refer to Appendix A in the supplement. Two reviewers (X.W. and E.L.) completed the literature search independently. In addition, these two reviewers further cross-checked the reference lists of all selected articles to identify other relevant studies. When screening discrepancies occurred, consensus was achieved after further discussion. This strategy was used to identify all of the articles included in previous reviews^26^.

### Eligibility criteria for considering studies for this review

We included studies that met the following inclusion criteria: (1) epidemiological and observational studies on congenital/infantile/childhood/pediatric cataracts that contained data on the population-based prevalence of CC or at least one epidemiological characteristic (laterality, morphology, associated comorbidities and etiology); (2) a diagnosis of CC made prior to the age of 18 years; (3) a diagnosis of CC based on the judgment of qualified pediatricians or ophthalmologists or on medical records showing a diagnosis of congenital/infantile cataract according to the International Classification of Disease and Codes; and (4) available full-text articles written in English or Chinese (with an English version of the abstract).

We excluded studies for the following reasons: (1) they were abstracts from conferences, full texts without raw data, duplicate publications, letters, or reviews; (2) the cataract diagnosis was not based on objective examination or medical records and involved self-reported cases; and (3) they were published in languages other than English or Chinese.

Two reviewers (X.W. and E.L.) independently selected the studies for final inclusion on the basis of these criteria. Disagreements between the two reviewers were resolved and adjudicated by the senior author (H.L.).

### Data Extraction and Outcomes of Interest

Two authors (X.W. and E.L.) extracted data and compared the results; discrepancies were resolved by discussion. We did not contact the authors of the eligible studies for additional data. The primary outcome was the population-based prevalence of CC. Other outcomes included the proportions of major epidemiologically based subtypes, including laterality (bilateral, unilateral), morphology (total, nuclear, posterior subcapsular, anterior polar, lamellar, posterior polar, sutural, mixed, others/nonspecific), comorbidity (isolated, with ocular disorder, with systemic disorder), and etiology (hereditary, nonhereditary, idiopathic).

### Quality Assessment and Statistical Analysis

The methodological quality was assessed using the Newcastle-Ottawa Scale (NOS) recommended by the Agency for Healthcare Research and Quality (AHRQ), available at http://www.ohri.ca/programs/clinical_epidemiology/oxford.asp. This scale uses a star system to assess the quality of a study in three domains: selection of study groups; comparability of groups; and ascertainment of outcomes.

The population-based CC prevalence from 16 studies was calculated from the raw proportions, and 95% confidence intervals (CIs) were calculated using the Wilson method^27^. To calculate the pooled prevalence, logit transformation was performed in advance for the prevalence range (1/10,000 to 1/1,000). For pooled data, the I^2^ statistic was used to estimate heterogeneity and risk of bias, specifically publication bias, based on the Egger test. I^2^ values of 50% or more were considered to indicate substantial heterogeneity, and the random-effects model was then used; otherwise, the fixed-effects model was used^28^. Analyses were conducted using the functions for proportion and summary meta-analysis in R (version 3.2.1, The R Foundation, Vienna, Austria). Potential sources of heterogeneity were further investigated using visual inspection of the data, forest plots, bias assessment plots, and meta-regression analysis. Univariate analyses were conducted in Stata (version 10.0, Stata Corporation, College Station, TX, USA) to test the individual association of selected covariates with the pooled estimates, including study type (birth cohort or others), research period (before or after year 2000), sample size (less or more than 100,000), world region (China [developing countries] or the rest of the world [developed countries]), and age at diagnosis (birth to 1 year old or older than 1 year). Based on univariate analyses, subgroup analyses were performed, and a multivariate meta-regression model was developed based on age at diagnosis, sample size and research period to determine the amount of heterogeneity; this analysis was performed using R (version 3.2.1, The R Foundation, Vienna, Austria).

## Additional Information

**How to cite this article**: Wu, X. *et al*. Prevalence and epidemiological characteristics of congenital cataract: a systematic review and meta-analysis. *Sci. Rep.* **6**, 28564; doi: 10.1038/srep28564 (2016).

## Supplementary Material

Supplementary Information

## Figures and Tables

**Table 1:** Overall characteristics of the included studies.

First author		Year	Study design	Nation/region	Age at diagnosis	Study period (Time span: years)	Sample size	NOS
Population-based epidemiology studies (prevalence of congenital cataract available)								
1	Stewart-Brown, S. L.^29^	1988	National prospective birth cohort	UK	10 y	1970–1980 (10)	12,853	6
2	Bermejo, E.^11^	1998	National prospective birth cohort	Spain	0–3 d	1980–1995 (15)	1,124,654	5
3	SanGiovanni, J. P.^4^	2002	National prospective birth cohort	USA	0–7 y	1959–1965 (6)	53,639	6
4	Haargaard, B.^30^	2004	National prospective birth cohort	Denmark	0–18 y	1980–2000 (20)	2,616,439	5
5	Rahi, J. S.^6^	2001	National surveillance	UK	0–15 y	1981–1996 (15)	734,000	5
6	Wirth, M. G.^19^	2002	National retrospective case review	Australia	NA	1977–2002 (25)	1,870,000	5
7	Hu, D. N.^31^	1987	National screening	China	NA	1980–1987 (7)	207,319	5
8	Stayte, M.^32^	1993	Regional prospective cohort	UK	0–5 y	1984–1989 (5)	6,687	5
9	Stoll, C.^12^	1997	Regional prospective cohort	France	At birth	1979–1994 (15)	212,479	4
10	Abrahamsson, M.^16^	1999	Regional surveillance	Western Sweden	NA	1980–1999 (19)	377,334	5
11	Bhatti, T. R.^15^	2003	Regional surveillance	Atlanta, USA	0–1 y	1968–1998 (30)	982,128	5
12	Nie, W. Y.^33^	2008	Regional screening	China	2–7 d	NA	15,398	3
13	Chen, Z.^34^	2014	Regional screening	Zhengzhou, China	3–6 y	2012–2013 (1)	9,246	6
14	Yang, L.^35^	2014	Regional screening	Hebei, China	6–14 y	2011–2012 (1)	6,299	5
15	Pi, L. H.^2^	2012	Regional cross-sectional field survey	Chongqing China	6–15 y	2006–2007 (1)	3,079	5
16	Holmes, J. M.^10^	2003	Regional retrospective case review	Upper mid-west USA	0–1 y	1978–1997 (19)	33,021	5
17	Halilbasic, M.^36^	2014	Regional retrospective case review	Tuzla Canton, Bosnia, Herzegovina	0–14 y	2003–2010 (17)	38,133	5
Hospital-based epidemiological studies								
18	Lawan, A.^37^	2008	Hospital-based cross-sectional study	Nigeria	0–10 y	2001–2005 (4)	4,163	3
19	Lin, H.^13^	2014	Hospital-based cross-sectional study	Guangzhou, China	0–18 y	2005–201 0(5)	136,154	5
Congenital/pediatric cataract-based case reviews								
20	Jain, I. S.^1^	1983	Congenital cataract case review	North India	NA	NA	76	2
21	Eckstern, M.^38^	1996	Congenital cataract case review	South India	0–15 y	1993–1994 (2)	514	4
22	Rahi, J. S.^18^	2000	Congenital cataract case review	UK	0–15 y	1995–1996 (1)	243	4
23	Haargaard, B.^20^	2004	Congenital cataract case review	Denmark	0–17 y	1977–2001 (24)	1,027	4
24	Johar, S. R.^14^	2004	Congenital cataract case review	West India	10 d-15 y	2001–2002 (1)	172	4
25	Perucho-Martinez, S.^21^	2007	Pediatric cataract case review	Spain	0–2 y	1986–2004 (18)	79	4
26	Lim, Z.^22^	2010	Pediatric cataract case review	California, USA	NA	1992–2002 (10)	778	4
27	You, C.^23^	2011	Pediatric cataract case review	Shandong, China	3 m-12 y	1995–2006 (11)	196	4

**Table 2:** Quality assessment of the included studies based on Newcastle-Ottawa Scale (NOS).

First Author	Selection				Comparability	Outcome			Scores
	Representativeness of exposed cohort	Selection of non -exposed cohort	Ascertainment of exposure	Outcome present at start of study	Comparability of cohorts	Assessment of outcome	Length of follow-up	Adequacy of follow-up	
Population-based epidemiology studies (prevalence of congenital cataracts available)									
Stewart-Brown, S. L.^29^	★	NA	★	★	NA	★	★	★	6
Bermejo, E.^11^	★	NA	★	★	NA	★	★	NA	5
SanGiovanni, J. P.^4^	★	NA	★	★	NA	★	★	★	6
Haargaard, B.^30^	★	NA	★	★	NA	★	★	NA	5
Rahi, J. S.^6^	★	NA	★	★	NA	★	★	NA	5
Wirth, M. G.^19^	★	NA	★	★	NA	★	★	NA	5
Hu, D. N.^31^	★	NA	★	★	NA	★	★	NA	5
Stayte, M.^32^	★	NA	★	★	NA	★	★	NA	5
Stoll, C.^12^	★	NA	★	NA	NA	★	★	NA	4
Abrahamsson, M.^16^	★	NA	★	NA	NA	★	★	★	5
Bhatti, T. R.^15^	★	NA	★	★	NA	★	★	NA	5
Nie, W. Y.^33^	★	NA	NA	NA	NA	★	★	NA	3
Chen, Z.^34^	★	NA	★	★	NA	★	★	★	6
Yang, L.^35^	★	NA	★	★	NA	★	★	NA	5
Pi, L. H.^2^	★	NA	★	★	NA	★	★	NA	5
Holmes, J. M.^10^	★	NA	★	★	NA	★	★	NA	5
Halilbasic, M.^36^	★	NA	★	★	NA	★	★	NA	5
Hospital-based epidemiology studies									
Lawan, A.^37^	★	NA	★	NA	NA	★	NA	NA	3
Lin, H.^13^	★	NA	★	★	NA	★	★	NA	5
Congenital/pediatric cataract case reviews									
Jain, I. S.^1^	★	NA	NA	NA	NA	★	NA	NA	2
Eckstern, M.^38^	★	NA	★	NA	NA	★	★	NA	4
Rahi, J. S.^18^	★	NA	★	NA	NA	★	★	NA	4
Haargaard, B.^20^	★	NA	★	NA	NA	★	★	NA	4
Johar, S. R.^14^	★	NA	★	NA	NA	★	★	NA	4
Perucho-Martinez, S.^21^	★	NA	★	NA	NA	★	★	NA	4
Lim, Z.^22^	★	NA	★	NA	NA	★	★	NA	4
You, C.^23^	★	NA	★	NA	NA	★	★	NA	4

**Table 3:** Pooled prevalence of CC considering different diagnosed age and quality scores.

Age diagnosed (Yeas old)	Quality Scores	Studies	Children	Pooled prevalence (1/10,000)	95% CI (%)	Heterogeneity (I2) (%)	95% CI (%)
0–1	≥7 (7–11)	4	2,517,137	1.91	[0.92; 3.97]	97.9	[96.5; 98.8]
0–1	0–11	6	2,745,014	2.61	[1.45; 4.69]	97.2	[95.7; 98.2]
0–18	≥7 (7–11)	8	3,326,875	3.42	[1.91; 6.12]	98.2	[97.5; 98.7]
0–18	0–11	17	8,302,708	4.24	[31.6; 51.9]	97.2	[96.5; 97.8]

**Table 4:** Random-effects model pooled proportion estimates by major CC epidemiological characteristics.

	Studies	Children	Pooled prevalence (%)	95% CI (%)	Heterogeneity (I2)	95% CI (%)
Laterality						
Bilateral	13	3,646	54.3	[45.9; 62.6]	96.0	[94.5; 97.1]
Unilateral	13	3,646	45.4	[36.6; 54.1]	96.4	[95.1; 97.4]
Morphology						
Total	6	1,393	31.2	[13.8; 48.7]	97.9	[96.9; 98.6]
Nuclear	8	2,478	27.2	[16.9; 37.4]	96.7	[95.1; 97.8]
Posterior subcapsular	4	2,082	26.8	[10.8; 42.8]	98.3	[97.3; 99.0]
Anterior polar	6	1,481	17.7	[8.04; 27.4]	97.2	[95.7; 98.2]
Lamellar	4	1,138	10.9	[4.45; 17.3]	75.0	[30.6; 91.0]
Posterior polar	3	2,023	7.25	[4.49; 10.0]	75.6	[19.4; 92.6]
Sutural	1	58	5.17	\	\	\
Mixed	3	1,212	16.8	[7.54; 26.0]	85.5	[58.4; 95.1]
Others/nonspecific	4	1,186	16.2	[6.60; 25.7]	91.5	[81.5; 96.1]
Comorbidity						
Isolated	5	1,586	62.3	[54.5; 70.2]	85.0	[66.6; 93.2]
With ocular disorder	6	2,716	22.7	[17.1; 28.2]	87.1	[74.3; 93.5]
With systemic disorder	6	1,885	17.3	[13.8; 20.7]	63.1	[10.6; 84.8]
Etiology						
Hereditary	9	2,430	22.3	[16.1; 28.4]	91.3	[85.8; 94.7]
Nonhereditary	5	1,675	11.5	[4.65; 18.3]	92.9	[86.3; 96.3]
Idiopathic	7	1,994	62.2	[57.2; 67.1]	74.1	[44.7; 87.9]
