# Supplementary material for: Prevalence and epidemiological characteristics of congenital cataract: a systematic review and meta-analysis
Source: Sci Rep. 2016 Jun 23;6:28564. doi: 10.1038/srep28564 (PMC4917826; doi:10.1038/srep28564)
Supplement: Supplementary Information [file srep28564-s1.pdf]

Supplementary information

Title Page

Prevalence and epidemiological characteristics of congenital cataract: a systematic review and meta-analysis

Xiaohang Wu, Erping Long, Haotian Lin[§], Yizhi Liu[§]

Institution: State Key Laboratory of Ophthalmology, Zhongshan Ophthalmic Center, Sun Yat-sen University, Guangzhou, Guangdong, 510060, People's Republic of China

[§]**Co-corresponding authors**: Yizhi Liu, M.D., Ph.D., Email: yizhi_liu@aliyun.com; Haotian Lin, M.D., Ph.D., Email: gddlht@aliyun.com; Address: Zhongshan Ophthalmic Center, Xian Lie South Road 54#, Guangzhou, China, 510060. Telephone number: +86-020-87330493, Fax: +86-020-87333271.

Appendix A. Search Strategy

PubMed Search Strategy

1.(((((cataract[Title/Abstract]) OR eye[Title/Abstract]) OR blindness[Title/Abstract]) OR visual impairment[Title/Abstract])

2.((((pediatric[Title/Abstract]) OR congenital[Title/Abstract]) OR infantile[Title/Abstract]) OR childhood[Title/Abstract]) OR children[Title/Abstract])

3.(((epidemiology[Title/Abstract]) OR prevalence[Title/Abstract]) OR incidence[Title/Abstract])

Final PubMed search

(1 AND 2) AND (3).
